# Protein-based Radiopharmaceuticals that target fibroblast activation protein alpha: a review of current progress

**DOI:** 10.1186/s41181-025-00356-5

**Published:** 2025-06-21

**Authors:** Abdelrahman Homedan, Darpan N. Pandya, Nicholas J. Schnicker, Thaddeus J. Wadas

**Affiliations:** 1 Department of Radiology, University of Iowa, Iowa City, IA 52242 USA; 2 Protein and Crystallography Facility, University of Iowa, Iowa City, 52242 USA; 3 Department of Molecular Physiology and Biophysics, University of Iowa, Iowa City, 52242 USA

**Keywords:** Fibroblast activation protein, Positron emission tomography, Single photon emission computed tomography, Cancer, Radiotherapy, Theranostics, Single domain antibody, Antibody, Antibody fragment

## Abstract

**Background:**

Fibroblast activation protein alpha (FAP) is a serine protease that is expressed at basal levels in benign tissues but is overexpressed in a variety of pathologies, including cancer. Consequently, significant research efforts have been expended to develop diagnostic radiopharmaceuticals and effective radiotherapies that target this protein. The aim of this review is to summarize the current progress on the development of protein-based radiopharmaceuticals that target FAP.

**Main body:**

A literature survey spanning nearly 40 years was conducted to assess the historical development and current progress in protein-based radiopharmaceuticals that target FAP. To date, more than 20 publications have been introduced that describe these agents in preclinical and clinical settings. This review summarizes the development and evaluation of radiopharmaceuticals involving antibodies, antibody fragments, and single domain antibodies.

**Conclusion:**

The results of this literature review demonstrate that while significant research efforts have been expended on peptide-based radiopharmaceuticals and small molecule FAP inhibitors, the development of protein-based radiopharmaceuticals that target FAP remains an active research area that has yet to reach its full potential.

## Background

**Fig. 1:**
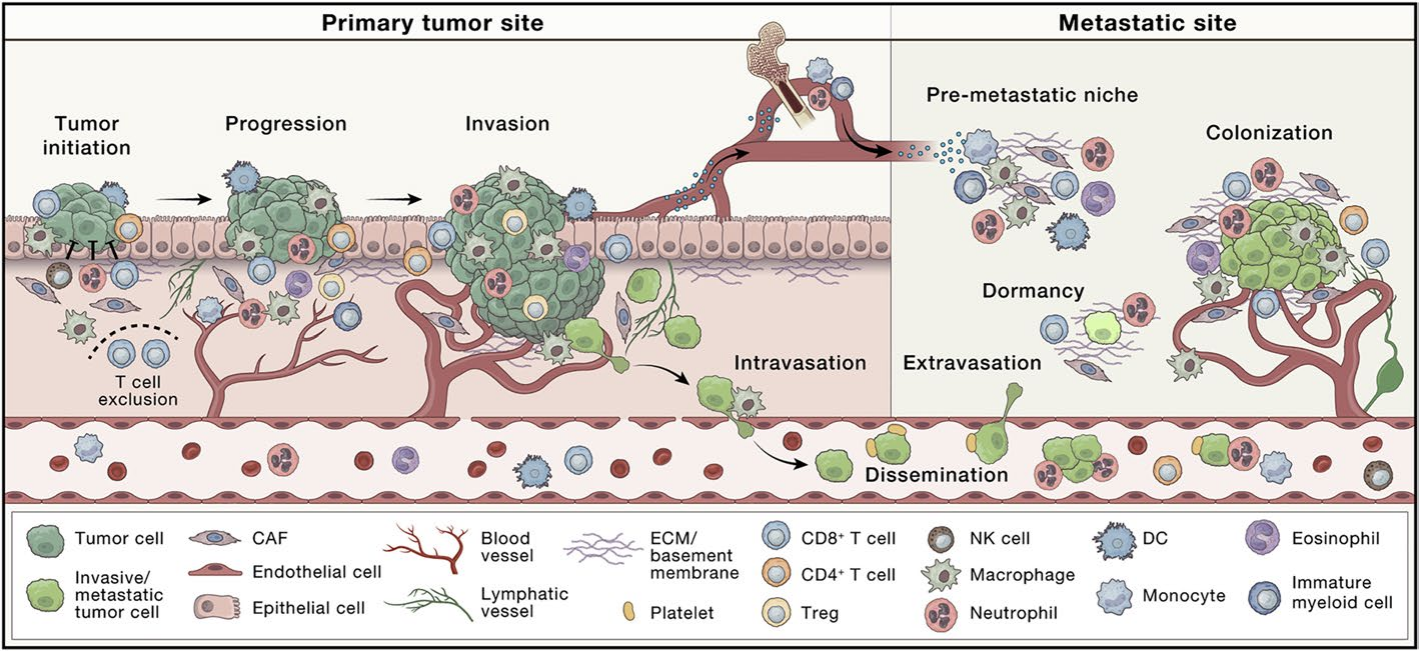
Microenvironmental regulation of primary tumor progression and metastasis The evolving tumor microenvironment (TME) during all stages of cancer progression is depicted with key representative cell types shown. The TME includes diverse immune cells, cancer-associated fibroblasts (CAFs), endothelial cells, and the extracellular matrix (ECM), among others. These components may vary by tissue type and co-evolve with the tumor as it progresses. The normal tissue microenvironment can constrain cancer outgrowth through the suppressive functions of immune cells, fibroblasts, and the ECM. However, for cancer to advance, it must evade these functions and instead influence cells in the TME to become tumor promoting, resulting in increased proliferation, invasion, and intravasation at the primary site. Cells and factors of the TME also play a vital role in preparing the premetastatic niche, regulating cancer cell survival in the circulation, and promoting extravasation. During the metastatic stages, the TME helps to control metastatic cell dormancy, emergence from this state, and subsequent metastatic outgrowth. This research was originally published in *Cancer Cell*. de Visser et al. The evolving tumor microenvironment: From cancer initiation to metastatic outgrowth. Cancer Cell 2023; 41:374-403. Copyright Cell Press

Cancer is a major cause of death worldwide and does so through the ability of malignant cells to leave the primary tumor and spread to other parts of the body via a complex process of metastasis (Hanahan 2022; Hanahan, et al. 2000; Hanahan, et al. 2011). Whether as part of a primary tumor or a metastatic deposit, cancer cells survive by creating a favorable microenvironment that facilitates their growth (de Visser, et al. 2023). This tumor microenvironment (TME) (de Visser, et al. 2023; Biray Avci, et al. 2024), or stroma, consists of extracellular matrix components (ECM) such as collagen and support cells such as immune cells (Li, et al. 2024; Lin, et al. 2025; Liu, et al. 2025; Moura, et al. 2025), nerve cells (Dragomir, et al. 2020; Gysler, et al. 2021; Wang, et al. 2020), and fibroblasts (Zhang et al. 2024a) whose normal functions have been corrupted to support tumor development through metabolic reprogramming (Hashimoto, et al. 2024; Hu, et al. 2024; Lin, et al. 2024; Liu, et al. 2024; Zhang et al. 2024b; Zhou, et al. 2024; Wang, et al. 2024), immunosuppression (Li, et al. 2024; Zhang et al. 2024a; Zhang, et al. 2024; Wang, et al. 2024; Guo, et al. 2023; Wang, et al. 2015; Zhu, et al. 2023; Chen, et al. 2017; Jia, et al. 2024; Jonker, et al. 2024; Lim 2024; Moinuddin, et al. 2024; Moraly, et al. 2024; Qian, et al. 2024; Shukla, et al. 2024), or dysregulated vascularization (Zheng, et al. 2022) and ECM remodeling (Fig. 1) (Song, et al. 2014). Over the last several decades scientists and clinicians have increasingly appreciated the contributions of the TME to cancer development and have long sought to understand the cells involved in tumor-stroma signaling and how to exploit these paracrine and juxtracrine signaling mechanisms for therapeutic development (Busek, et al. 2018). One population of stromal cells that has continued to receive attention are tumor associated fibroblasts (TAFs), which are also referred to as cancer-associated fibroblasts (CAFs) (Chen, et al. 2024; Cui, et al. 2024; Kamali Zonouzi, et al. 2022; Kawasaki, et al. 2024; Lv, et al. 2024; Raaijmakers, et al. 2024; Saude-Conde, et al. 2024; Wen, et al. 2025; Yin, et al. 2024). They have been of continued interest since, unlike cancer cells, they are not considered to be transformed genetically, are perceived to be resistant to therapy-induced clonal selection, and differ from normal fibroblasts found in benign adult tissues in their morphology and gene expression profile (Scott, et al. 2003). However, research has demonstrated that they contribute to tumor development, progression, and metastasis by degrading the extracellular matrix, establishing disordered fibrovascular networks that enable rapid tumor growth, and contributing to immunosuppression. Thus, significant research effort has been expended to identify biomarkers on CAFs that can be exploited therapeutically to improve clinical outcomes for cancer patients.

One biomarker that has received considerable attention is fibroblast activation protein alpha (FAP), which is a 170 kDa, type II transmembrane serine protease that is part of the dipeptidyl peptidase (DPP) family; it is involved with ECM remodeling and displays both dipeptidyl peptidase and gelatinase/collagenase activity (Aggarwal, et al. 2008; Edosada, et al. 2006a; Edosada, et al. 2006b; Jacob, et al. 2012). It is structurally composed of a 6 amino acid cytoplasmic tail, a 20 amino acid transmembrane domain, and a 734 amino acid extracellular domain. Interestingly, FAP’s biological functions are not fully defined, and as a result, the scientific community’s understanding of FAP’s structure, cellular localization, and ability to contribute to mechano- and intra-cellular signaling within healthy and diseased tissues continues to evolve. Although there is a widely held view that FAP expression is typically low to undetectable in most normal healthy tissue, data does suggest that there are varying levels of FAP expression in certain types of normal tissues and in the plasma of healthy donors (Roberts, et al. 2013; Busek, et al. 2016a; Liao, et al. 2017; Ackermann, et al. 2016). FAP is also found to be transiently expressed in healing wounds and in many non-oncological diseases in which activated fibroblasts are present, such as rheumatoid arthritis and different types of fibrosis.

From an oncology perspective, FAP expression has been observed in some sarcomas and neuroepithelial tumors (Yuan, et al. 2013; Mentlein, et al. 2011; Rettig, et al. 1988). Additionally, it has been observed in 90% of epithelial cancers (Rettig, et al. 1988; Dolznig, et al. 2005; Garin-Chesa, et al. 1990; Mathew, et al. 1995; Niedermeyer, et al. 1997; Rettig, et al. 1986; Rettig, et al. 1993), including breast (Cremasco, et al. 2018; Dendl, et al. 2023; Huang, et al. 2011; Jia, et al. 2014; Jung, et al. 2015; Tchou, et al. 2013), ovary (Berkovitz-Shperling, et al. 2024; Corvigno, et al. 2025; Lai, et al. 2012; Li, et al. 2020; Mhawech-Fauceglia, et al. 2015), lung (Novruzov, et al. 2024; Chen, et al. 2022; Yang, et al. 2024), skin (Huber, et al. 2003), and pancreas (Hashimoto, et al. 2024; Zhu, et al. 2023; Cheng, et al. 2022; Zhao, et al. 2023), but the majority of this expression has been associated with CAFs located within the stroma. Like its role in normal tissues, the role of FAP in cancer development continues to be refined with it being implicated in promoting tumor cell proliferation, migration (Monsky, et al. 1994), and invasion through its influence on a variety of cellular pathways including I3K/AKT (Wang, et al. 2014a), RAS/ERK (Wang, et al. 2014b), SHH/GLI (Jia, et al. 2018), and FAK (Jia, et al. 2014), and through ECM remodeling (Huang, et al. 2011), which may contribute to a reduction in ECM stiffness that improves tumor cell mobility while contributing to the establishment of cytokine, chemokine, and matrikine gradients, which produce an immunosuppressive and tumor promoting environment (Chen, et al. 2017; Yang, et al. 2016). Pathologically, increased FAP expression has been positively correlated with higher tumor grade and worse overall survival across many cancers, although its prognostic value varies depending on the tumor’s grade and type (Chen, et al. 2017; Jung, et al. 2015; Mhawech-Fauceglia, et al. 2015; Wang, et al. 2014a; Yang, et al. 2016; Gong, et al. 2014; Park, et al. 2015; Park, et al. 2016; Kawase, et al. 2015; Liu, et al. 2015; Lopez, et al. 2016; Errarte, et al. 2016; Miao, et al. 2014; Wen, et al. 2017; Hu, et al. 2017).

**Fig. 2:**
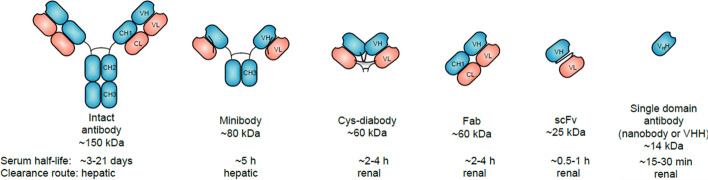
Human and Camelid antibody fragments. Structures of human antibody fragments discussed in this review. Heavy chain is colored in blue and light chain in red. Antibody domains are labelled with their appropriate names used for molecular imaging. Heavy chain is colored in blue and light chain in red. Antibody do-(CH—constant heavy; VH—variable heavy; VL—variable light; VHH—variable heavy of heavy-chain-only antibodies). Size and circulation half-lives are mentioned below the structures. This research was originally published in Biomolecules. Berland, l. et al. Nanobodies for Medical Imaging: About Ready for Prime Time? Biomolecules 2021; 11:637. Copyright MDPI

Over the last few decades, numerous attempts to create effective cancer therapies through FAP targeting have been documented; these include vaccines (Shahvali, et al. 2023; Shin, et al. 2023), inhibitors (Cortesi, et al. 2025; Hu, et al. 2005; Jansen, et al. 2013; Jansen, et al. 2014; Ryabtsova, et al. 2012; Tran, et al. 2007; Wu, et al. 2010), chimeric antigen receptor T-cell therapy (Rurik, et al. 2022; Wang, et al. 2014b), monoclonal antibodies (mAbs) (Sun, et al. 2024; Yang, et al. 2021a), and nanodrugs (Shin, et al. 2023; Hadinoto, et al. 2013; Zhou, et al. 2021; Xin, et al. 2021; Abbasi, et al. 2024). Recently, the nuclear medicine community has developed a strategy that targets FAP to deliver diagnostic and therapeutic radionuclides to tumors, which has resulted in numerous publications describing a large library of radiolabeled FAP inhibitors and peptides. However, since these reports have been covered thoroughly in several excellent reviews (DiMagno, et al. 2023; Gilardi, et al. 2022; Roustaei, et al. 2022; Windisch, et al. 2020; Yang, et al. 2021b; Baum, et al. 2024; Mori, et al. 2023a; Mori, et al. 2023b), this review will discuss the historical development and theranostic potential of monoclonal antibodies, antibody fragments, and single domain antibodies that target FAP (Fig. 2).

## Main text

### Antibody-based radiopharmaceuticals that target FAP

Rettig and coworkers were the first to produce the F19 mAb from mice immunized with lung fibroblasts and describe its interaction with the F19 antigen, which was later renamed fibroblast activation protein (Rettig, et al. 1986). A subsequent investigation using the F19 mAb revealed that FAP’s antigen expression profile varies across different types of cells, tissues, and tumors (Rettig, et al. 1988). The cultured cell map was created using 12 established sarcoma cell lines and various normal fibroblast cultures obtained from 20 individuals. The normal tissue map was created using a diverse collection of normal adult and fetal tissues. The tumor tissue map was created using a collection of 200 malignant tumors. Comparison of these maps allowed researchers to identify patterns of glycoprotein expression unique to certain types of sarcomas, neuroectodermal tumors, carcinomas, and lymphomas. This enabled them to classify tumors into subsets based on their antigen profiles, providing insights into their origins, behaviors, and potential treatment strategies. With respect to FAP, the team observed that the F19 glycoprotein was rarely expressed in most normal adult tissues with a few exceptions, including on a small number of fibroblasts and on the alpha cells of the pancreatic islets. However, FAP was found to be more broadly expressed in fetal mesenchymal tissues. More importantly, FAP was found to be expressed in many different types of sarcoma tissue, including fibrosarcomas, malignant fibrous histiocytomas, leiomyosarcomas, osteosarcomas, chondrosarcomas, and liposarcomas, but not expressed in those with a “small round cell” phenotype. Although the vast majority of neuroectodermal tumors, carcinomas, and lymphomas were found to be FAP negative, expression was observed in the reactive stroma that surrounds many of these tumors, suggesting that FAP has a role in mesenchymal activation. Further expanding on these observations, Retting and coworkers then examined additional tissue to understand FAP^+^ fibroblast induction and expression (Garin-Chesa, et al. 1990). Notably, FAP^+^ fibroblasts were expressed in scar tissue following surgical incisions and in the reactive mesenchyme of primary and metastatic epithelia tumors including colorectal, breast, ovarian, bladder, and lung. In contrast, benign tumors such as fibroadenomas demonstrated only moderate FAP^+^ fibroblast populations, suggesting that the high FAP expression in the former tumors reflects a phenotype that may be a valuable target for therapeutic intervention.

Given its potential as a therapeutic target, Rettig and coworkers turned their attention to studying the interaction and binding characteristics of the F19 mAb with FAP (Rettig, et al. 1994). Using the F19 mAb and several other mAbs, the team was able to deduce that FAP exists as a 95 kDa subunit, which was named FAPα that contains the F19 mAb binding epitope, and a non-covalently bound subunit that was described as FAPβ that lacks the F19 mAb binding epitope. Based upon these analyses, the team was confident that any therapeutic development involving the F19 mAb would result from an interaction with the alpha isoform. Historically, these studies represent initial significant contributions to the emerging research discipline of cancer immunology that offered the first valuable insights into differential FAP expression in normal tissues, malignant tissues, and cultured cells, paving the way for future therapeutic development involving the F19 mAb and its derivatives.

Not long after these reports, a team at the Memorial Sloan Kettering Cancer Center translated these initial findings into a Phase 0 clinical trial (Welt, et al. 1994). In the first study, Welt et al. used the chloramine T method to label the F19 mAb with Iodine-131 (^131^I: β^−^—emitter: t_1/2_ = 8._0_ d; E _β_^−^_max_ = 0.97 MeV) to prepare [^131^I]I-F19 mAb in order to determine if the radiolabeled antibody could detect FAP expression in colorectal carcinoma tumors. Seventeen subjects with hepatic metastases from colorectal carcinoma were intravenously administered the radiopharmaceutical at three different dose levels (0.2-mg (7 patients), 2-mg (4 patients), and 8-mg (6 patients)) approximately one week before their scheduled tumor resection or insertion of a hepatic artery catheter for regional chemotherapy. Subjects underwent planar scintigraphy (PS), single photon emission computed tomography (SPECT), and computed tomography (CT) scanning after infusion. Serum samples were taken throughout the course of the week to determine the radiopharmaceutical’s pharmacokinetic profile, and tissue biopsies were obtained at the time of clinical intervention, which was completed as part of each subject’s treatment plan.

Based upon the trial results, the radiopharmaceutical was well-tolerated by subjects since no administration-related toxicities were reported at any dose level, but as anticipated, human anti-mouse IgG and IgM antibody responses were observed 2–6 weeks after administration. Pharmacokinetically, the agent was observed to behave similarly to other radiolabeled mAbs and had a T_1/2_ of 26 ± 17 h, which allowed the research team to deduce that the optimal imaging time was a 3–5-day window after infusion. These pharmacokinetic properties, coupled with the limited expression profile of FAP, yielded significant tumor-to-serum and tumor-to-liver ratios of 9:1 and 21:1, respectively. As expected, SPECT imaging was more sensitive than PS, with the former being able to detect liver lesions as small as 1 cm in diameter. However, clear distinctions of detection efficiency could not be obtained from the small cohort of subjects. For example, several subjects had surgically confirmed hepatic lesions that were only identified with [^131^I]I-F19 mAb SPECT and not CT. In another subject though, [^131^I]I-F19 mAb SPECT failed to identify all hepatic lesions while the CT and MRI scans were equivocal. These discrepancies in detection were ascribed to differences in cancer progression and tumor biology of each subject and the amount of reactive stroma within each lesion. This led the authors to conclude that given the limitations of scanner technology at the time, the best approach for tumor detection was to use [^131^I]I-F19 mAb SPECT imaging to complement traditional CT or MRI imaging methods that were being used as standard of care.

In an extension of these studies, Tanswell et al. investigated the population pharmacokinetics by combining the data from a Phase I trial involving metastatic colorectal carcinoma patients with additional [^131^I]I-F19 mAb imaging data involving metastatic sarcoma patients who enrolled in a second, separate Phase I trial (Tanswell, et al. 2001). The serum concentration–time data of [^131^I]I-F19 mAb from study participants was obtained after co-infusion of the F19 mAb and radiopharmaceutical with colorectal carcinoma subjects administered a 0.2 mg dose (n = 4) or a 2 mg dose (n = 3) of the F19 mAb, while all subjects with soft tissue sarcoma were administered a 1 mg dose of the anti-FAP antibody. Using the combined data from both studies, pharmacokinetic analysis revealed that the volumes derived from the imaging and serum data correlated well, suggesting minimal distribution of the monoclonal antibody in FAP^−^ tissues. While these results supported the results obtained in previous studies, the high variability between individual cohort pharmacokinetics, the exclusion of patients with comorbidities, the pooling of subjects from two separate studies that examined two separate cancer types with variable FAP expression, and the variability in dosing were assessed as complicating aspects of this experimental design and weakened the clinical impact of this report.

Although [^131^I]I-F19 mAb demonstrated initial success in detecting metastatic hepatic lesions in colorectal cancer patients, the human-anti-mouse antibody response observed in many of the subjects demonstrated that modifications to the antibody would be required if additional clinical studies were to continue. As a result, Scott and coworkers generated sibrotuzumab, a humanized version of the F19 mAb (Scott, et al. 2003; Riechmann, et al. 1988; Verhoeyen, et al. 1988). Humanization was achieved using the complementarity determining region grafting strategy with preservation of selected framework region residues, and all preclinical in vitro research demonstrated that sibrotuzumab bound FAP in the same manner as the F19 mAb; sibrotuzumab did not inhibit the serine protease’s enzymatic activity and could be radiolabeled with ^131^I using the chloramine-T method without disrupting affinity for its FAP binding site. These positive results led to the initiation of a phase I open-label dose-escalation trial involving 15 male and 11 female subjects with either colorectal carcinoma or non-small cell lung cancer (Scott, et al. 2003). Approximately six subjects were enrolled to receive sibrotuzumab at each dose level (5 mg/m^2^, 10 mg/m^2^, 25 mg/m^2^, and 50 mg/m^2^). Each received 12 weekly infusions with dose escalation occurring after any one subject received four consecutive weeks of treatment without exhibiting signs of a dose limiting toxicity (DLT). In addition to these therapeutic doses, 8–10 mCi (296–370 MBq/infusion) of [^131^I]I-sibrotuzumab was prepared and co-administered with the first three infusions. Gamma camera imaging was conducted five times between the initial infusion and day seven of the study to help researchers understand the safety, immunogenicity, and pharmacokinetics of this new mAb. Over the course of this trial, gamma camera imaging revealed that [^131^I]I-sibrotuzumab’s blood circulation time was consistent with other radiolabeled mAbs and that it did not extensively bind to non-target tissues in vivo. Additionally, elevated, rapid, and selective uptake of [^131^I]I-sibrotuzumab was observed in tumors larger than 1.5 cm in all subjects as early as 2 days post-infusion, suggesting to the research team that the humanized mAb reliably targeted FAP^+^ tumors with high specificity. However, serum analysis revealed a dose-dependent pharmacokinetic profile with clearance rates decreasing as the infused doses escalated, suggesting that it was cleared through a mechanism which becomes overwhelmed at higher mAb concentrations in the blood pool. This led the authors to speculate that this may increase the potential for toxicity and adverse off-target effects. Safety was investigated by measuring subjects’ human anti-human antibody (HAHA) responses and monitoring adverse events related to the mAb infusion. Out of the 25 patients given two or more infusions of sibrotuzumab, HAHA responses occurred in 8 of them (32%), and the study team was able to correlate these responses with altered mAb biodistribution, including increased liver uptake, reduced tumor uptake, and faster drug clearance. Additionally, adverse events including anorexia, flushing, back pain, fever, rigors, hypertension, coughing, dyspnea, and hypoxia were observed in six subjects throughout the study. However, only 1 subject, who was administered 50 mg/m^2^ of the mAb, experienced a common terminology criteria for adverse events (CTCAE) grade 3 back pain episode, which was considered a DLT due to the trial design (Le-Rademacher, et al. 2020; Chen, et al. 2010). All other subjects experienced mild grade 1 or grade 2 adverse events that were well managed during the study. As a result, a maximum tolerated dose was not reached, and these findings led the researchers to conclude that sibrotuzumab was safe for patients suffering from FAP^+^ cancers. However, the continued immunogenicity issue surrounding this mAb proved to be an effective barrier to its widespread clinical use.

Considering the lack of therapeutic efficacy and other limitations observed with the F19 mAb and its derivatives (Tahtis, et al. 2003), Fischer and coworkers sought to develop anti-FAP mAbs with improved properties such as high affinity for the FAP protein and rapid cellular internalization (Fischer, et al. 2012). Using phage display, the team isolated the Fab fragments ESC11 and ESC14, which were observed to bind to human and murine FAP protein. The research team then engineered these fragments into full length IgG1 mAbs so that avidity would be increased during the mAb-FAP interaction. Each agent was modified with CHX-A”-DTPA and radiolabeled with Lutetium-177 (^177^Lu^3+^: β^−^—emitter: t_1/2_ = 6.7d; E _β_^−^_max_ = 0.497 MeV). The ^177^Lu versions of F19 and A33 mAbs were prepared as control agents, and the potential of the ESC11 and ESC14 radiopharmaceuticals were assessed using SPECT/CT imaging, efficacy studies, and the FAP^+^ and FAP^−^ melanoma cell lines SK-MEL-187 and SK-MEL-16, respectively. When comparing the four radiopharmaceuticals in vitro, researchers observed that [^177^Lu]Lu-CHX-A”-DTPA-ESC11 was internalized by the SK-MEL-187 tumor cells at high levels and in a specific manner but displayed low uptake in the SK-MEL-16 tumor cells when compared to the other three radiopharmaceuticals. In vivo, [^177^Lu]Lu-CHX-A”-DTPA-ESC11 achieved elevated accumulation in FAP^+^ tumors but not in tissues with basal FAP expression at 72 h p.i. It also outperformed [^177^Lu]Lu-CHX-A”-DTPA-ESC14 and [^177^Lu]Lu-CHX-A”-DTPA-F19, which both exhibited lower tumor and higher spleen accumulations. Comparative therapy studies using all four radiopharmaceuticals demonstrated that when compared to the isotype control radiopharmaceutical [^177^Lu]Lu-CHX-A”-DTPA-A33, which produced a median cohort survival of 32 d, all three anti-FAP antibodies delayed tumor growth, with the median survival of animals receiving [^177^Lu]Lu-CHX-A”-DTPA-F19 being 32 d, and greater than 43 d for [^177^Lu]Lu-CHX-A”-DTPA-ESC14 and [^177^Lu]Lu-CHX-A”-DTPA-ESC11. Despite these promising results, further studies to assess the applicability of ESC11-based radiotherapy or improve on the original design never appeared in the literature.

**Fig. 3:**
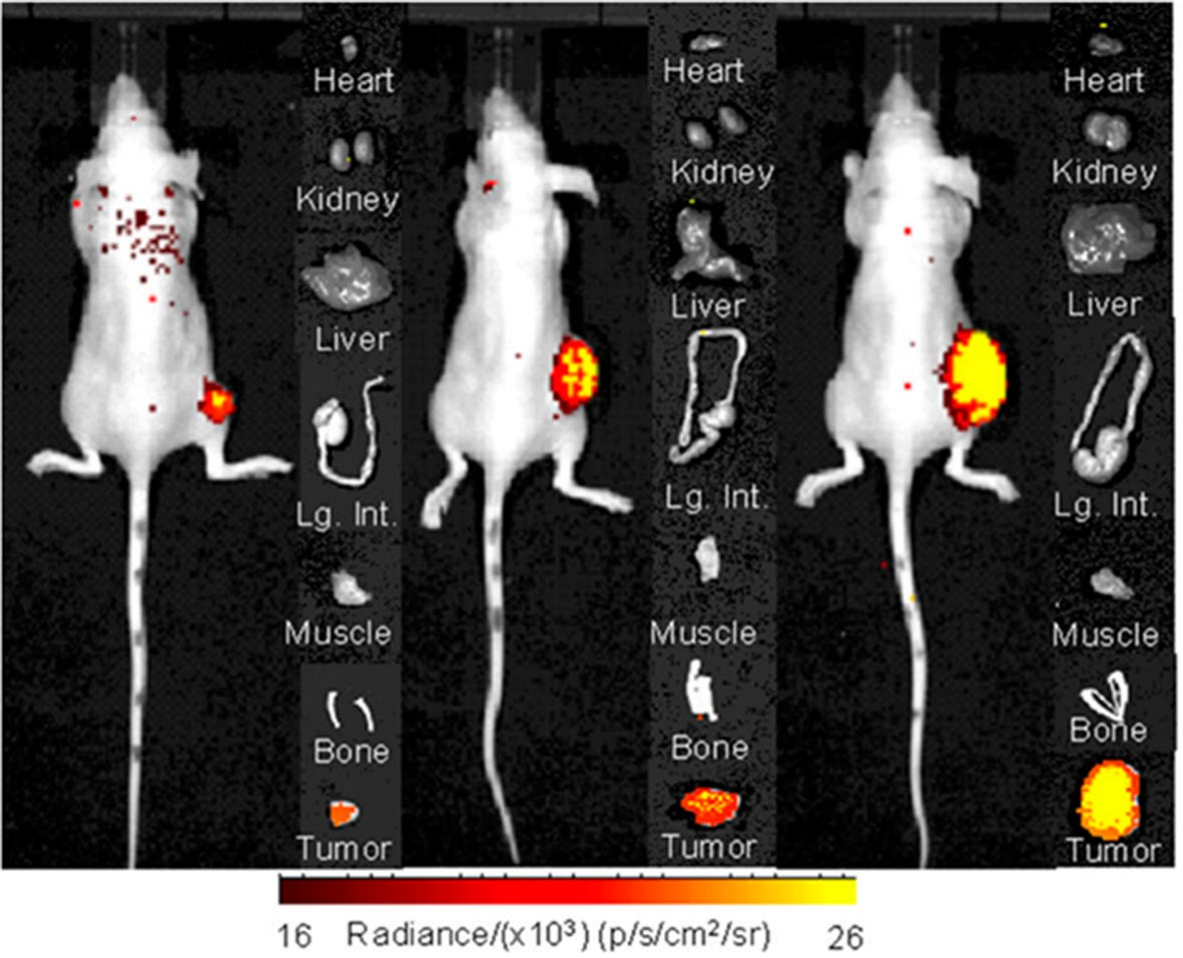
Cerenkov luminescence image of FAP^+^U87MG tumors-bearing mice 72 h p.i. using [^89^Zr] Zr-DFO-Bz-F19. Experiments indicated increased luminescence intensity among the FAP ^+^ tumors. FAP^-^ tissues such as muscle displayed an average radiance not exceeding background levels. Ex vivo organ imaging confirmed the in vivo results. Based on ROI analysis, FAP ^+^ tumors had an average radiance of 8.5 x_ 10^3^ _ ± 1.5 _x 10^3^ p/s/cm^2^/sr. This research was originally published in Molecules. Pandya, D. et al. Imaging of Fibroblast Activation Protein Alpha Expression in a Preclinical Mouse Model of Glioma Using Positron Emission Tomography. Molecules 2020; 25:3672. Copyright MDPI

More than a decade later, Pandya et al. took advantage of the nuclear decay properties of zirconium-89 (^89^Zr: t_½_ = 78.4 h, β^+^: 22.8%, E_β+max_ = 901 keV; EC: 77%, E_γ_ = 909 keV) PET and Cerenkov Luminescence Imaging (CLI) to examine FAP expression in a murine model of glioblastoma multiforme (GBM), since it was previously reported that GBM tumors expressed FAP and its expression increased as tumor aggressiveness increased (Busek, et al. 2016a, b; Pandya, et al. 2020a). Further, the research team sought to study the relationships between small animal PET/CT and CLI, since at the time, CLI was a new and promising technology that combined nuclear medicine and optical imaging techniques that surgeons might have harnessed in the operating theater to detect and eradicate minimally residual disease, which is a confounding factor in GBM surgical management (Bhatt, et al. 2018a; Boykoff, et al. 2024; Chin, et al. 2013; Ciarrocchi, et al. 2017; Grootendorst, et al. 2016; Schwenck, et al. 2016; Xu, et al. 2011). Using [^89^Zr]Zr-DFO-Bz-F19 mAb and athymic nude mice bearing FAP^+^ U87MG tumors, the team performed conventional biodistribution studies, small animal PET/CT imaging, CLI, and autoradiography in conjunction with correlative histology as part of this work. Consistent with other radiolabeled antibodies reported in the literature, accumulation in the blood was high at 2 h p.i. (23.72 ± 2.92 %ID/g) but decreased significantly (11.11 ± 3.10 %ID/g) by 72 h p.i. Accumulation in the muscle slightly increased from 1.92 ± 0.35 %ID/g at 2 h p.i. to 1.97 ± 0.44 %ID/g at 72 h. [^89^Zr]Zr-DFO-Bz-F19 initially demonstrated modest accumulation in the tumor 2 h p.i. (5.94 ± 0.84 %ID/g), but increased significantly after 72 h (16.40 ± 3.65 %ID/g). This accumulation was reduced upon injection of F19 mAb blockade, suggesting that a specific interaction between the radiopharmaceutical and the protease was occurring in vivo. Without blockade, the average tumor-to-blood ratios were 0.24, 0.92, 1.2, and 1.4 at 2, 4, 24, 48 and 72 h p.i., respectively. Similarly, average tumor-to-muscle ratios of 3.1, 7.3, 7.2, and 8.3 were observed at 2, 4, 24, 48, and 72 h p.i., respectively. As a result, acceptable and increasing image contrast in the small animal PET/CT images and CLI images were reported as the time course of these experiments progressed (Fig. 3). Moreover, the team was able to validate radiopharmaceutical uptake with FAP expression using autoradiography and correlative histology. Despite these promising results, it was observed that overcoming the technical hurdles relating to detection sensitivity, which were associated with CLI at that time, would be required before CLI of FAP^+^ tumors could progress toward clinical investigation.

**Fig. 4:**
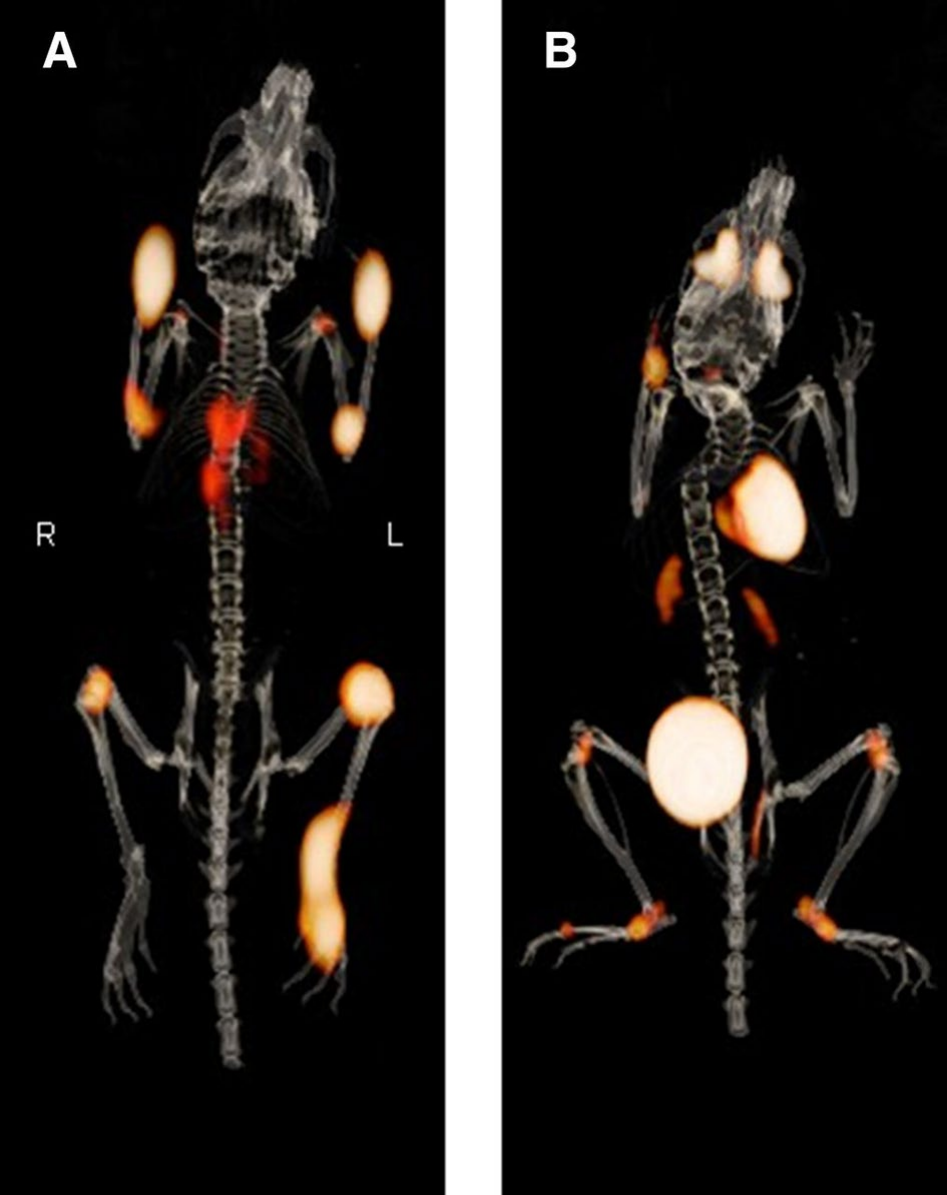
Small animal PET/CT imaging of CIA mice imaged with [^89^Zr] Zr-28H1. **A** PET/CT scans (3D) of mouse with CIA, injected with 5 MBq of [^89^Zr] Zr-28H1 and scanned at 72 h after injection. Joint scores were 1.75, 1.5, 0.25, and 2 (front right, front left, hind right, and hind left, respectively). **B** PET/CT scan of mouse with CIA, injected with 10 MBq of [^18^F] F-FDG and scanned at 1 h after injection. Joint scores were 0, 0.25, 1.25, and 0.25 (front right, front left, hind right, and hind left, respectively). This research was originally published in the Journal of Nuclear Medicine. Laverman, P. et al. Immuno-PET and Immuno-SPECT of Rheumatoid Arthritis with Radiolabeled Anti-Fibroblast Activation Protein Antibody Correlates with Severity of Arthritis. The Journal of Nuclear Medicine 2015; 56: 778–83. Copyright Society of Nuclear Medicine and Molecular Imaging, Inc

In 2015 Laverman and coworkers exploited the upregulation of FAP on activated synoviocytes, which play a role in joint destruction and pannus formation as rheumatoid arthritis (RA) progresses. To study this, the team used PET and SPECT to determine if the fully human anti-FAP IgG1 antibody 28H1 could detect RA-based inflammation and joint destruction and predict disease severity in a murine model of collagen-induced arthritis (CIA) (Laverman, et al. 2015). As part of these studies, the team immunized male DBA/1 J mice with bovine collagen type II to illicit a CIA phenotype that replicates rheumatoid arthritis in humans. Approximately 30 days after immunization, the animals underwent SPECT/CT imaging with [^111^In]In-28H1 or the isotype control radiopharmaceutical, [^111^In]In-DP47GS. Further, disease severity was independently measured using an arthritis score, which was based upon symptomatic observation such as redness, swelling, and ankylosis. SPECT/CT imaging revealed elevated uptake of [^111^In]In-28H1 in the inflamed joints, rather than in the surrounding tissues, which diminished by 72 h p.i. Contrarily, [^111^In]In-DP47GS demonstrated less uptake and retention in inflamed joints, suggesting to the team that [^111^In]In-28H1 possessed greater specificity towards the FAP antigen than the latter agent. A strong correlation between arthritis scores and uptake of [^111^In]In-28H1 in mouse joints was observed, but this correlation was not observed with [^111^In]In-DP47GS. Similar results were observed when PET/CT imaging was conducted using [^89^Zr]Zr-28H1 and [^89^Zr]Zr-DP47GS (Fig. 4). However, greater retention of these radiopharmaceuticals was observed in the bones of mice that were not involved with the arthritic phenotype. This suggested to the authors that either FAP-mediated binding and retention in the bone marrow was occurring or that transchelation of ^89^Zr from the radiopharmaceutical to the bone matrix was occurring in vivo. While the former issue would be difficult to overcome due to normal biology, recent innovations in ^89^Zr chelation strategies would, if combined with this antibody, improve this radiopharmaceutical’s utility. (Bhatt, et al. 2018b; Bhatt, et al. 2018c; Pandya, et al. 2017; Pandya, et al. 2019; Pandya, et al. 2020b). Subsequent studies demonstrated that derivative radiopharmaceuticals ^99m^Tc-S-HYNIC-28H1 and [^111^In]In-DTPA-28H1 were able to non-invasively monitor the effects of RA therapy in the CIA mouse model (van der Geest, et al. 2017; Terry, et al. 2016), and since that time, this mAb has been investigated in additional clinical scenarios involving FAP-related disease processes (Boswinkel, et al. 2023).

Another example of creative FAP targeting was reported by Hintz et al., who described the characterization of a single-chain variable fragment (scFv) which was isolated from a naïve murine scFv phage display library and later engineered to be a full-length chimeric IgG monoclonal antibody designated B12-IgG (Hintz, et al. 2019). In subsequent publications, the research team prepared the immuno-PET agent [^89^Zr]Zr-B12-IgG and used it to evaluate FAP expression in several models of prostate cancer (Hintz, et al. 2020). Using quantitative image analysis and a flank xenograft model which consisted of a PCa cell line engineered to overexpress FAP, the authors were able to demonstrate that the amount of radioactivity in the FAP^+^ tumors of animals receiving the radiopharmaceutical was threefold higher than in the tumors of animals receiving the isotype control radiopharmaceutical, [^89^Zr]Zr-ICC-IgG, at 72 h p.i. Additionally, the study team was able to demonstrate that FAP targeting could be achieved in an intratibial bone metastasis model. In this model, [^89^Zr]Zr-B12-IgG accumulation was observed to be fivefold greater when compared to the inoculated limbs of animals receiving the isotype control radiopharmaceutical. While these studies demonstrate that FAP^+^ PCa primary tumors and metastatic disease could be visualized with [^89^Zr]Zr-B12-IgG, it should be acknowledged that the use of engineered cell lines or the use of an intratibial bone metastasis model may not accurately reflect the development and progression of PCa in a clinical setting. Further testing with additional animal models that more accurately reflect clinical disease would pave the way for clinical evaluation in prostate cancer patients.

Recently, Xu and coworkers evaluated the anti-FAP mAb, PKU-525, which the authors described as a sibrotuzumab derivative (Xu, et al. 2023a). In their first report, the authors generated the radiopharmaceutical [^89^Zr]Zr-DFO-PKU525 and the beta therapy agent [^177^Lu]Lu-DOTA-NCS-PKU525. Both agents were evaluated in HT1080 cells that were engineered to overexpress FAP. Biodistribution, PET/CT, and SPECT/CT studies demonstrated appreciable tumor uptake and retention over the time course of these experiments. Imaging contrasts and tumor-to-tissue ratios were consistent with the in vivo behavior of other radiolabeled anti-FAP mAbs; contrast and ratios improved as the radiopharmaceutical extravasated from the blood pool and began to localize in target tissues. Radiotherapy studies with [^177^Lu]Lu-DOTA-NCS-PKU525 demonstrated meaningful tumor growth control in animals bearing HT1080-FAP^+^ tumors, while significant toxicity was not reported by the authors. A subsequent publication by the same group examined the potential of the actinium-225 (^225^Ac^3+^: α^++^—emitter: t_1/2_ = 10 d; E_αmax_ = 6–8 MeV)-based radiopharmaceutical in a 4T1 breast cancer cell line that was engineered to overexpress FAP (Song, et al. 2023). As in the former publication, biodistribution studies demonstrated a reasonable pharmacokinetic profile while therapy studies revealed meaningful tumor growth control. While these studies show promise for the PKU525 mAb in FAP theranostics development, several limitations of this work were noted without being directly addressed in either publication. Foremost, the authors never described how the PKU525 ligand differs from sibrotuzumab, nor did they provide a rationale of the modifications to the ligand and how those modifications influenced the latter’s pharmacokinetics. Secondly, the cell lines used in both studies were engineered to overexpress FAP and consequently, may not accurately reflect a realistic clinical situation. Additionally, while the therapy studies were successfully conducted, rigorous toxicity studies were not completed as part of the study plan. Toxicity studies are particularly important in the case of systemic radiotherapies since it is well known that latent toxicity can occur months after treatment (Tafreshi, et al. 2021; Kristiansson, et al. 2024; Alattar, et al. 2023; Bertoni, et al. 2023). Finally, the authors also chose to compare therapeutic efficacy of their agent with ^225^Ac-FAPI-46, an FAP inhibitor, but it is not clear why this comparison was chosen in these studies. A more rigorous comparison would have been to compare the ^225^Ac-PKU525 radiotherapy with a ^225^Ac-radiolabeled version of sibrotuzumab or similar mAb. Thus, while the data reported within these two publications holds promise, the compelling shortfalls in study design require tempered enthusiasm when considering future clinical translation.

### Single domain antibodies that target FAP

Single domain antibodies (sdAbs), also referred to as VHHs or nanobodies, are a unique type of immunoglobulin that was first discovered in the sera of *Camelidae* such as camels, alpacas, and llamas (Fig. 2) (Yang, et al. 2020; Berland, et al. 2021). When compared to conventional heavy chains (VH) of regular IgG molecules, their three complementarity determining regions (CDRs) are enlarged to provide a greater surface area for antigen interactions, making them well-suited for binding restricted sites such as cavities or sterically hindered epitopes (Khodabakhsh, et al. 2018; Jovcevska, et al. 2020). Moreover, they contain additional hydrophilic amino acids within the conserved framework region. Unlike monoclonal antibodies, VHH proteins have a variety of unique characteristics, which have made them promising ligands for diagnostic imaging agent and therapy development (Jin, et al. 2023). Their small size results in improved tumor penetration and in some cases allows them to cross the blood–brain barrier. They retain high affinity and specificity for their target antigens, with low off-target accumulation since they are belived to bind their target antigen in several orientations (Ketaren, et al. 2025). Furthermore, compared to the stability exhibited by a conventional antibody, they are unexpectedly robust due to their high refolding capacity, being able to recover from chemical denaturation with minimal damage to functionality. While several reports have demonstrated that they can tolerate environmental conditions associated with radiochemistry including high temperatures, elevated pressures, and non-physiological pHs (Xu, et al. 2025) some publicactions suggest these characteristics may be sdAb-specific (Akazawa-Ogawa, et al. 2014; Kunz, et al. 2018) and have prompted additional research into improving their stability in vitro and in vivo (Kunz, et al. 2018; Dingus, et al. 2022; Zhong, et al. 2021). Additionally, sdAbs are simple and inexpensive to produce on the milligram scale in a laboratory setting since they lack post-translational modifications and can be synthesized in microbial, plant, or synthetic systems (Wu 2025; Muyldermans 2021; Liu, et al. 2022). As a result, the last three decades have witnessed explosive growth in research evaluating them as diagnostic and therapeutic agents for a variety of pathologies. As of 2020, there were over 15 clinical trials involving sdAbs (Yang, et al. 2020; Yang, et al. 2022).

To address the barriers associated with monoclonal antibodies, Xu et al. developed anti-FAP nanobodies by immunizing alpacas with human, recombinant FAP, generating a focused library, and selecting proteins for affinity towards the FAP protein (Xu, et al. 2023b). From a phage library, two VHHs were selected, isolated, and engineered to include a human IgG4 Fc region to prolong their serum half-lives and enhance their tumor penetration abilities. The two constructs, identified as AMS002-1-Fc and AMS002-2-Fc, were derivatized with DFO (deferoxamine) and DOTA (1,4,7,10-tetraazacyclododecane-N,N',N″,N‴-tetraacetic acid) to facilitate ^89^Zr and ^177^Lu radiochemistry, respectively.

In an initial evaluation, researchers prepared [^89^Zr]Zr-DFO-AMS002-1-FC and [^89^Zr]Zr-DFO-AMS002-2-FC and used small animal PET/CT to compare the biodistributions in mice bearing FAP^+^ HT1080 xenografts. Based upon the imaging data, [^89^Zr]Zr-DFO-AMS002-1-FC demonstrated a better pharmacokinetic profile and yielded better tumor-to-blood and tumor-to-muscle ratios when compared to [^89^Zr]Zr-DFO-AMS002-2-FC, leading the authors to further scrutinize the former agent more rigorously in vitro and in vivo. In vitro, immunofluorescence imaging using FAP^+^ HT1080 and FAP^−^ A549 cells revealed the increased accumulation of Cy7-AMS002-1-Fc in FAP^+^ HT1080 but not in FAP^−^ A549 cells. Further, cellular uptake and internalization studies using the same cell lines and [^89^Zr]Zr-DFO-AMS002-1-FC corroborated the immunohistochemistry results. Additionally, biodistribution and small animal PET/CT studies using [^89^Zr]Zr-DFO-AMS002-1-FC were conducted in a mouse model bearing FAP^+^ HT1080 tumors and FAP^−^ A534 tumors that were grown in contralateral flanks. The biodistribution studies demonstrated that [^89^Zr]Zr-DFO-AMS002-1-FC accumulation was much higher in the FAP^+^ HT1080 xenografts than in the FAP^−^ A549 xenografts, suggesting that the accumulation of the radiopharmaceutical in the HT1080 tumors was due to a specific mAb-FAP interaction, corroborating the results obtained from the in vitro cell studies.

Considering the promising imaging results obtained with [^89^Zr]Zr-DFO-AMS002-1-FC, the research team decided to explore the therapeutic efficacy of [^177^Lu]Lu-DOTA-AMS002-1-FC in mice bearing FAP^+^ HT1080 tumor xenografts. In these studies, cohorts of tumor bearing animals received an injection of saline, [^177^Lu]LuCl_3_, or the radiotherapy. Throughout the study, animals receiving the radiotherapy did not demonstrate adverse effects from the treatment and had tumors that were significantly smaller than the tumors of animals receiving either saline or [^177^Lu]LuCl_3_.

Although these findings are interesting, the therapeutic studies lacked a control cohort that was treated with the non-radioactive AMS002-1-Fc. Excluding this control cohort limited the ability to distinguish the therapeutic effects of ^177^Lu’s radiative decay and any therapeutic effects that would be attributed to a pharmacological action of the protein alone. Further, while [^177^Lu]LuCl_3_ was used as a control agent to distinguish the effects of nonspecific radiotoxicity from targeted therapy, a more appropriate control would have been to treat a cohort of animals with a ^177^Lu-labeled agent that is not specific for FAP. Doing so would have allowed for a more rigorous evaluation of [^177^Lu]Lu-DOTA-AMS002-1-FC’s ability to exert a therapeutic effect. Finally, a proper toxicity study was not undertaken to determine the maximum tolerated dose in these radiotherapy studies, which limits the impact of the reported findings.

Dekempeer et al. explored the potential of anti-FAP sdAbs for diagnosing and treating cancer (Dekempeneer, et al. 2023). In their study, single domain antibodies were identified from a phage display library that was derived from B cell mRNA, which was isolated from alpacas immunized against human and murine recombinant FAP. After multiple rounds of selection and characterization, researchers chose to further study the 4AH29 sdAb since it bound mouse FAP (muFAP) and human FAP (huFAP), did not bind to human dipeptidyl peptidase-4 (DPPIV), which is the closest protein homolog of FAP in humans, and displayed picomolar binding affinity to a conserved epitope on FAP that was distinct from its catalytic site.

**Fig. 5:**
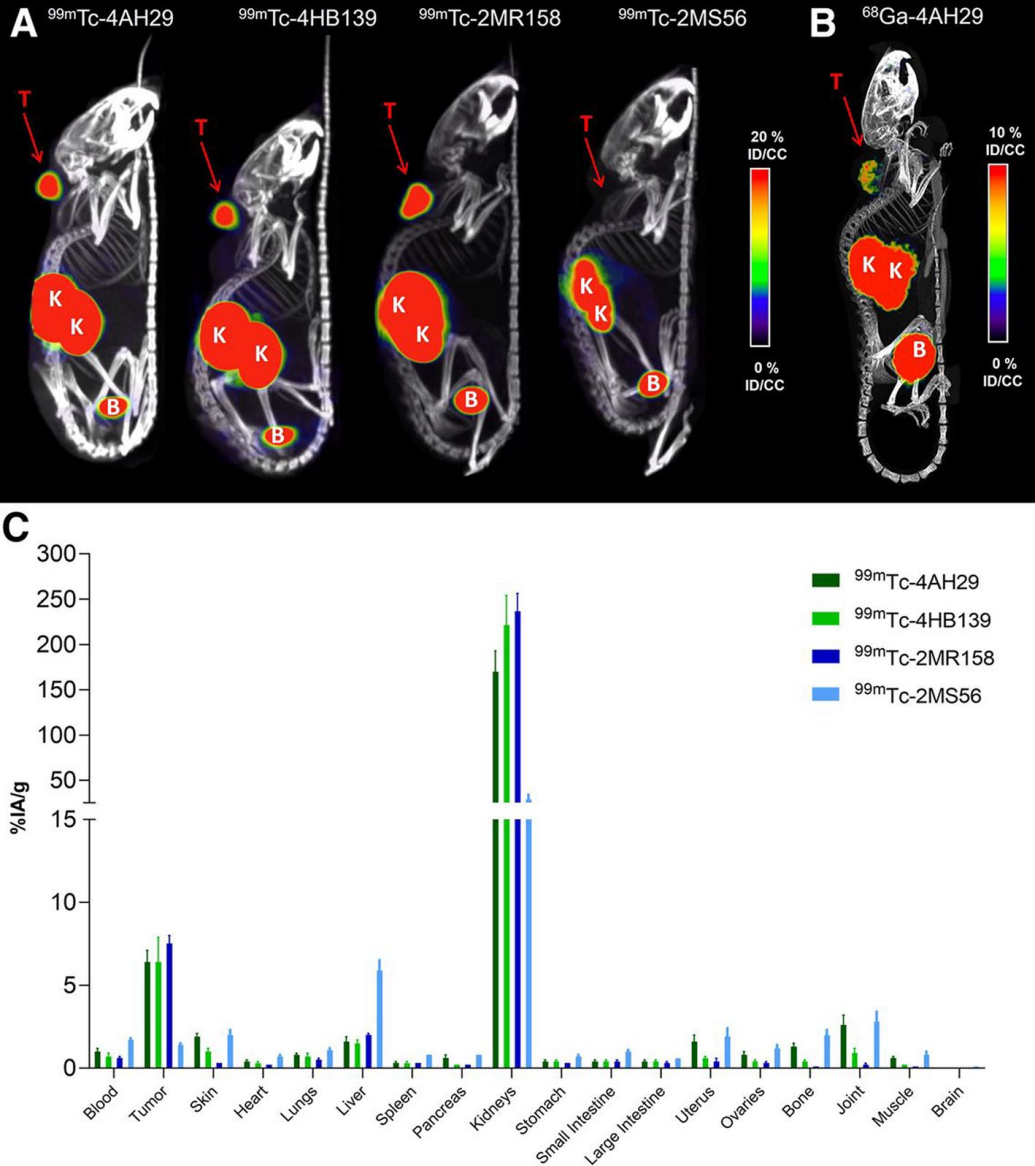
Biodistribution of ^99m^Tc- and ^68^Ga-labeled anti-FAP sdAbs. **A** Maximum intensity projections (MIPs) corresponding to small-animal SPECT/CT images obtained 1 h after administration of ^99m^Tc-labeled sdAbs in hFAP-positive U87-MG tumor–xenografted mice (n = 3). **B** MIPs corresponding to small-animal PET/CT image 1 h after administration of [^68^Ga]Ga-DOTA-4AH29 in hFAP-positive U87-MG tumor–xenografted mice (n = 3). %ID/CC = percentage injected activity per cubic centimeter; B = bladder; K = kidney; T = tumor. **C** Ex vivo biodistribution obtained after dissections 1.5 h after administration of ^99m^Tc-labeled sdAbs. This research was originally published in The Journal of Nuclear Medicine. Dekempeer, Y. et al. Preclinical Evaluation of a Radiotheranostic Single-Domain Antibody Against Fibroblast Activation Protein α. The Journal of Nuclear Medicine 2023; 64: 1941–1948. Copyright Society of Nuclear Medicine and Molecular Imaging

To understand the theranostic potential of the sdAb 4AH29, it was conjugated with guanidinomethyl iodobenzoate (GMIB) and radiolabeled with ^131^I for SPECT imaging. It was also radiolabeled with technetium-99 m (^99m^Tc: γ- emitter: t_1/2_ = 6.0 h; E_γmax_ = 0.14 MeV) through the His6-Tag previously engineered into the protein (Fig. 5). The research team also conjugated it to DOTA to facilitate radiolabeling with either gallium-68 (^68^Ga: β + emitter: t_1/2_ = 68 min; E_β+max_ = 1.92 MeV) for PET imaging or ^225^Ac for targeted alpha particle therapy (TAT). The radiolabeled variants [^68^Ga]Ga-DOTA-4AH29 and [^131^I]I-GMIB-4AH29 were used to image the FAP^+^ U87MG tumors implanted in mice. Small animal PET/CT and SPECT/CT revealed rapid and robust uptake of [^68^Ga]Ga-DOTA-4AH29 and [^131^I]I-GMIB-4AH29, respectively, in U87MG tumor xenografts. This was not observed in the images of tumor bearing animals that were injected with the control sdAb radiopharmaceuticals [^131^I]I-GMIB-R3B23 or [^68^Ga]Ga-DOTA-R3B23. SPECT/CT imaging with ^99m^Tc-4AH29 in the same animal model also revealed high specificity and high tumor uptake, but elevated radioactivity levels were also observed in kidney tissue at 1 h p.i. However, biodistribution data that was collected at later time points with [^131^I]I-GMIB-4AH29 demonstrated that the radioactivity was actively being excreted over the time course of the experiment.

The therapeutic potential of 4AH29 radiolabeled with the β-emitting ^131^I and the α-emitting ^225^Ac radiometal were also evaluated in biodistribution studies. While [^131^I]I-GMIB-4AH29 and [^225^Ac]Ac-DOTA-4AH29 accumulated in tumors after injection, the retention of radioactivity was also elevated in kidney tissue. However, kidney excretion in mice receiving [^131^I]I-GMIB-4AH29 appeared to be more efficient than that observed in animals injected with [^225^Ac]Ac-DOTA-4AH29, which was probably due to the retention of ^225^Ac’s daughter products within renal tubule cells.

**Fig. 6:**
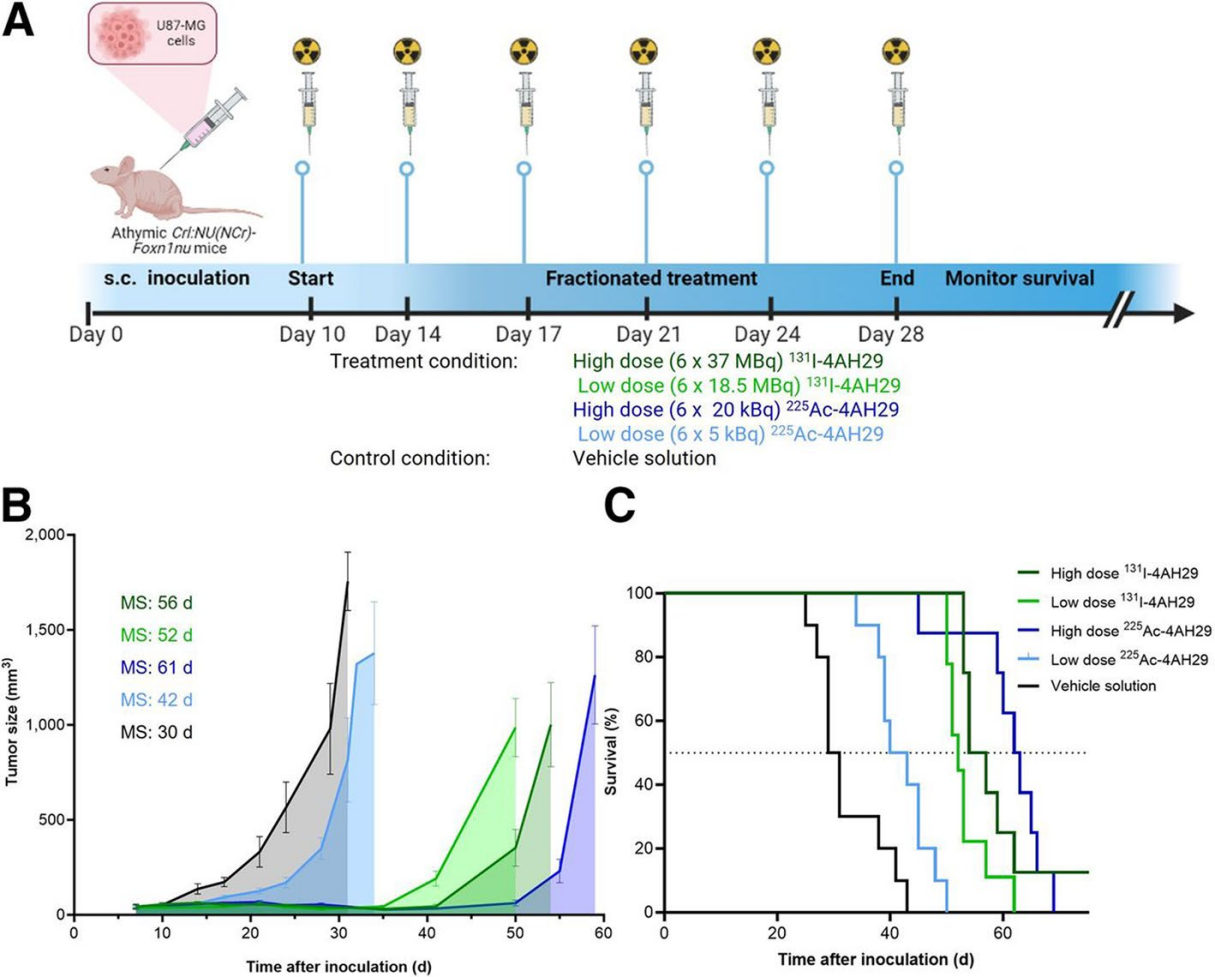
Therapeutic efficacy of [^131^I]I-GMIB-4AH29 and [^225^Ac]Ac-DOTA-4AH29. **A** Therapeutic potential of [^131^I]I-GMIB-4AH29 and [^225^Ac]Ac-DOTA-4AH29 was evaluated in U87-MG tumor–bearing mice (n = 10). **B** Subcutaneous tumor development over time for each treatment group. **C** Kaplan–Meier survival curve obtained after administration of [^131^I]I-GMIB-4AH29 or [^225^Ac]Ac-DOTA-4AH29. MS = median survival; s.c. = subcutaneous. This research was originally published in The Journal of Nuclear Medicine. Dekempeer, Y. et al. Preclinical Evaluation of a Radiotheranostic Single-Domain Antibody Against Fibroblast Activation Protein α. The Journal of Nuclear Medicine 2023; 64: 1941–1948. Copyright Society of Nuclear Medicine and Molecular Imaging

To further understand the utility of these agents, toxicity studies were completed with [^131^I]I-GMIB-4AH29 and [^225^Ac]Ac-DOTA-4AH29 using a fractionated, 6-dose scheme where each animal received one-sixth of the total dose over an 18-day period (Fig. 6). The study team chose five different dose cohorts of 0, 27.5, 55.5,111,166.5, and 222 MBq for [^131^I]I-GMIB-4AH29 studies and 0,15, 30, 60,120, and 240 kBq for [^225^Ac]Ac-DOTA-4AH29 studies. Regardless of the dose, all mice injected with [^131^I]I-GMIB-4AH29 survived the 180-day study with minimal weight loss, and only mild or moderate changes in kidney architecture were observed at the highest doses of 166.5 MBq or 222 MBq, respectively. Contrastingly, mice injected with [^225^Ac]Ac-DOTA-4AH29 exhibited late-stage toxicity, a 20% weight reduction, and reduced survival at the three highest doses. For animals receiving [^225^Ac]Ac-DOTA-4AH29 at the cumulative dose levels of 240, 120, and 60 kBq, the mean survival was 129, 138, and 150 days, respectively, and all mice injected with cumulative doses of 30 and 15 kBq survived the 180-day study. However, histological analysis revealed changes in the kidney structure of animals receiving doses as low as 30 kBq.

Once a dose-toxicity relationship had been established, the research team conducted therapy studies using the fractionated dose paradigm above but selected a high and low dose of both radiopharmaceuticals to be administered to tumor bearing animals. Once tumors were established, animals received a cumulative dose of 222 MBq or 111 MBq of [^131^I]I-GMIB-4AH29 or 120 kBq or 30 kBq of [^225^Ac]Ac-DOTA-4AH29 over an 18-day period. Data suggested that both the high and low doses provided tumor growth control and prolonged survival when compared to a saline treatment. The mean survival of mice injected with 120 kBq [^225^Ac] Ac-DOTA-4AH29 was 61 days, and 42 days for the mice injected with 30 kBq. The mean survival of mice injected with 222 MBq of [^131^I]I-GMIB-4AH29 was 56 days, and 52 days for the mice injected with the lower dose.

**Fig. 7:**
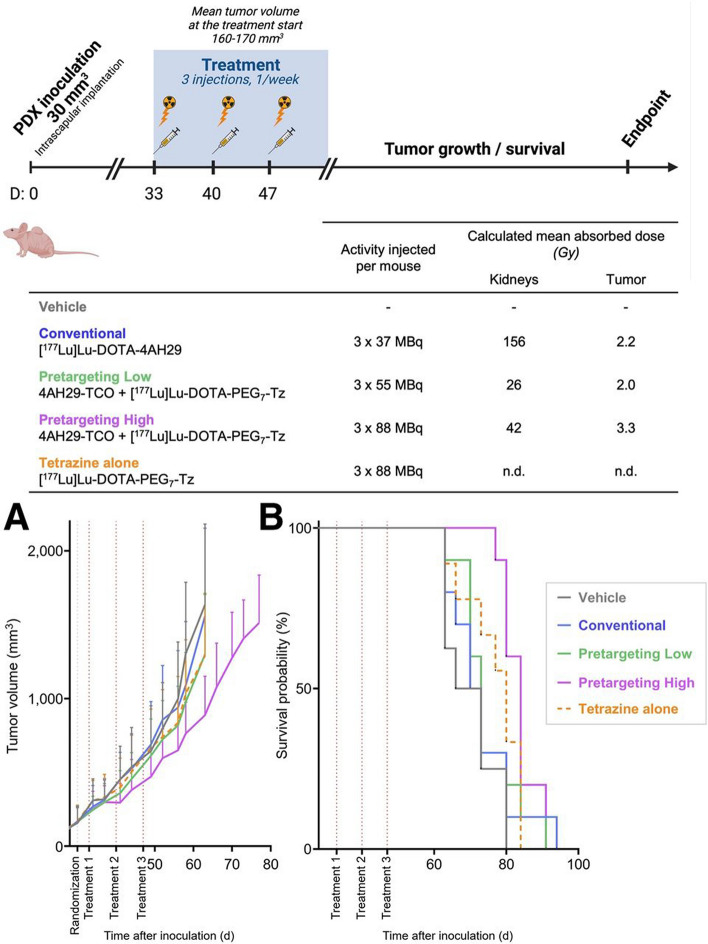
Therapy study in PDX2494-P5 with schematic overview of therapeutic schedule and dosimetry. (**A** and **B**) Tumor growth control (**A**) and Kaplan–Meier plot of survival probability (**B**) as function of time. Mice were euthanized when tumor volume was 2,000 mm^3^. Survival data reflect progression of primary tumors, because no adverse effects led to anticipated animal euthanasia. n.d. = not determined; Tz = tetrazine. This research was originally published in The Journal of Nuclear Medicine. Poty, S. et al. Optimizing the Therapeutic Index of sdAb-Based Radiopharmaceuticals Using Pretargeting. The Journal of Nuclear Medicine 2024; 65: 1564–1570. Copyright Society of Nuclear Medicine and Molecular Imaging

Considering the positive results from these therapy studies, the research team explored the use of the sdAb in conjunction with immunotherapy (Ceuppens, et al. 2025), generated additional PET agents (Dierick, et al. 2024), and investigated ways to mitigate the radiopharmaceutical accumulation in kidney tissues (Poty, et al. 2024). In these last studies, the team compared the biodistribution and therapeutic index (TI) of [^177^Lu]Lu-DOTA-4AH29 with a pre-targeting approach involving 4AH29-TCO and [^177^Lu]Lu-DOTA-PEG_7_-tetrazine. In proof of principal studies, the team demonstrated that an 8.5-fold reduction in mean absorbed dose to the mouse kidney could be achieved provided a gap of 8 h. was included between the injection of 4AH29-TCO and [^177^Lu]Lu-DOTA-PEG_7_-tetrazine. Furthermore, a fractionated therapy study, which involved FAP^+^ patient derived xenograft (PDX) tumor bearing animals was also completed (Fig. 7). This study involved animals receiving three separate doses of either saline, [^177^Lu]Lu-DOTA-4AH29 (37 MBq/dose), 4AH29-TCO and [^177^Lu]Lu-DOTA-PEG_7_-tetrazine (55 MBq/dose), 4AH29-TCO and [^177^Lu]Lu-DOTA-PEG_7_-tetrazine (88 MBq/dose) or [^177^Lu]Lu-DOTA-PEG_7_-tetrazine (88 MBq/dose). Studies revealed that pre-targeting combined with high dose administration provided sufficient tumor growth control over time, extended median overall survival, and demonstrated a kidney toxicity profile that was more consistent with that observed in the saline treated cohort. Although longitudinal toxicity studies are required to demonstrate that this approach is safer for kidney function, the positive data generated thus far bodes well for additional preclinical testing and clinical translation; the results of those studies are awaited eagerly.

## Conclusions

Although much about FAP biology remains unknown, several novel therapeutic approaches to combat cancer and other disease states by targeting FAP have been described in the literature. While much of the nuclear medicine community has focused on the development of small molecule inhibitors or peptides to deliver radioactivity to FAP^+^ tissues, a vibrant research effort to develop antibody-based agents that target FAP exists, which is evidenced by the many creative research efforts described in this review. These radiopharmaceuticals possess several key advantages over small molecules and peptides, but as with all diagnostic and therapeutic agents, additional research will be required to maximize their potential. It is expected that the integration of protein engineering and machine learning into the antibody discovery and development process will enable researchers to mitigate initial limitations in pharmacokinetics, immunogenicity, toxicity, or efficacy and lead the nuclear medicine community to renew its enthusiasm for clinical disease management using protein-derived theranostics that target FAP. 

## Data Availability

Not applicable.

## Abbreviations

%ID/g: Percent injected dose per gram of tissue
CAFs: Cancer associated fibroblasts
CDRs: Complementarity determining regions
CIA: Collagen-induced arthritis
CLI: Cerenkov luminescence imaging
CT: Computed tomography
CTCAE: Common terminology criteria for adverse events
DFO: Deferoxamine
DLT: Dose limiting toxicity
DOTA: 1,4,7,10-Tetraazacyclododecane-N,N',N″,N‴-tetraacetic acid
DPP: Dipeptidyl peptidase
DPPIV: Dipeptidyl peptidase-4
ECM: Extracellular matrix components
FAP: Fibroblast activation protein
GBM: Glioblastoma multiforme
GMIB: Guanidinomethyl iodobenzoate
HAHA: Human anti-human antibody
huFAP: Human FAP
mAb: Monoclonal antibody
muFAP: Mouse FAP
p.i.: Post-injection
PDX: Patient derived xenograft
PS: Planar scintigraphy
RA: Rheumatoid arthritis
scFv: Single-chain variable fragment
sdAb: Single domain antibody
SPECT: Single photon emission computed tomography
TAF: Tumor associated fibroblast
TAM: Tumor-associated macrophage
TAT: Targeted alpha-particle therapy
TI: Therapeutic index
TME: Tumor microenvironment
TMEM: TME of Metastasis
VH: Heavy chains
VHH: Variable heavy domain of heavy chain
